# Increased Prevalence of Celiac Disease in Patients with Cystic Fibrosis: A Systematic Review and Meta-Analysis

**DOI:** 10.3390/jpm11090859

**Published:** 2021-08-28

**Authors:** Marcell Imrei, Dávid Németh, Zsolt Szakács, Péter Hegyi, Szabolcs Kiss, Hussain Alizadeh, Fanni Dembrovszky, Piroska Pázmány, Judit Bajor, Andrea Párniczky

**Affiliations:** 1 Institute for Translational Medicine, Medical School, University of Pécs, Szigeti út 12., H-7624 Pécs, Hungary; marcell.imrei@gmail.com (M.I.); davidsum96@gmail.com (D.N.); szaki92@gmail.com (Z.S.); hegyi2009@gmail.com (P.H.); kissszabolcs1995@gmail.com (S.K.); dembrovszky.f@gmail.com (F.D.); pazmanyp11@gmail.com (P.P.); 2 János Szentágothai Research Centre, University of Pécs, Szigeti út 12., H-7624 Pécs, Hungary; 3 Division of Gastroenterology, First Department of Medicine, Medical School, University of Pécs, Szigeti út 12., H-7624 Pécs, Hungary; bajor.judit8@gmail.com; 4 Centre for Translational Medicine, Department of Medicine, University of Szeged, Tisza Lajos krt. 109., H-6725 Szeged, Hungary; 5 Doctoral School of Clinical Medicine, University of Szeged, Tisza Lajos krt. 109., H-6725 Szeged, Hungary; 6 Division of Hematology, First Department of Medicine, Medical School, University of Pécs, Szigeti út 12., H-7624 Pécs, Hungary; alizadeh.hussain@pte.hu; 7 Heim Pál National Pediatric Institute, H-1089 Budapest, Hungary

**Keywords:** cystic fibrosis, celiac disease, autoimmunity, antibody, prevalence

## Abstract

Objectives: Immune regulation seems to be altered in cystic fibrosis (CF), thus potentially predisposing patients to developing autoimmune diseases (AID). In this meta-analysis, we aimed to evaluate the prevalence of celiac disease (CeD) among CF patients as by far the most commonly reported autoimmune disease in this population and, secondly, to review the observations on other, less frequently studied autoimmune diseases. Methods: We conducted a systematic literature search for studies that discussed AIDs among CF patients. Following standard selection and data collection, we calculated pooled raw prevalence with 95% confidence intervals (CI) for biopsy-verified CeD and seropositivity. Results: Out of the 21 eligible studies, 15 reported on CeD. Pooled prevalence of biopsy-verified CeD was 1.8% (CI 1.1–2.7%) according to a homogeneous dataset from six prospective, consecutive screening studies, while it proved to be 2.3% (CI 1.1–4.7%) according to a heterogeneous dataset from the other studies. Tissue transglutaminase IgA positivity was detected in 4.5% of CF cases (CI 2.8–6.9%), while tissue transglutaminase IgA–endomysial antibody IgA double positivity was found in 2.4% of them (CI 1.5–3.9%). Findings on other AIDs were strongly limited. Conclusions: The pooled prevalence of CeD in CF seemed to be more than twice as high compared to the global prevalence; therefore, routine screening of CeD could be considered in CF.

## 1. Introduction

Cystic fibrosis (CF; OMIM: 219700) is an autosomal recessive disease caused by the loss-of-function mutation of the cystic fibrosis transmembrane conductance regulator (CFTR) gene encoding the CFTR protein [1,2]. CF is considered the most common lethal genetic disease among Caucasians, with a reported birth prevalence of 1:2000–1:4000 live births [3,4].

Life expectancies have improved remarkably to 40 years of age, while pulmonary and gastrointestinal (GI) manifestations are to be highlighted as being responsible for the majority of fatalities [5].

In addition to the epithelial cells being thought to be the most affected due to CFTR dysfunction, innate and adaptive immune systems might be compromised both in terms of quality and quantity of response [6,7,8]. Immunological imbalance often provides the basis for developing autoimmune diseases (AID) as comorbid conditions [9]. In CF, the most investigated AID is celiac disease (CeD). However, Goodchild and Taylor calculated that the chance of the coincidence of these two conditions was between 1:2,000,000 and 1:5,900,000 [10,11]. In the past 10 years, several case–control studies have been published, reporting a wide range (1.2–2.13%) of proven CeD incidence in European CF patients [12]. This comorbidity could be described as a vicious cycle of chronic intestinal mucosal damage due to pancreas insufficiency, malnutrition, intestinal inflammation, and slow GI motility, resulting in an overload of incompletely or undigested nutrients (e.g., proteins), leading to an immunological response to antigens (e.g., gluten) [13,14,15]. There is a growing interest that can be explained by a set of arguments including the following: (1) CeD is the most common AID with an expected prevalence of approximately 1% in the general population; (2) due to the GI manifestations of CF, it is difficult to decide whom to screen for CeD; and (3) CeD is treatable by prescribing a lifelong gluten-free diet, thus significantly improving quality of life.

The European CF Society (2018) recommends that CeD should be considered in children with poor growth, while the CF Foundation (2016) recommends that all providers should be aware of the presenting symptoms of CeD [16,17]. In line with this, the leading pediatric and adult CeD guidelines (released by the NASPGHAN, ESPGHAN, ACG, and ESsCD) do not mention CF as a condition that increases the risk of CeD [18,19,20,21]. Despite the different pathomechanisms, both CF and CeD cause malabsorption in the majority of cases; thus, in everyday practice, physicians often face the challenge of distinguishing between CF-related or potentially CeD-related GI symptoms. There is an unmet clinical need in understanding the prevalence in AIDs in CF with special focus on CeD.

In this work, we aimed to perform a meta-analysis to evaluate the prevalence of CeD among CF patients and, as a secondary objective, to provide a review of the prevalence studies about less frequently studied autoimmune diseases.

## 2. Materials and Methods

This meta-analysis is reported in accordance with the Preferred Reporting Items for the Systematic Reviews and Meta-Analyses (PRISMA) statement [22] (Table S1). We uploaded our protocol to the International Prospective Register of Systematic Reviews (PROSPERO) under the registration number CRD42020155862. Originally, we planned to use two risk of bias assessment tools for the quality assessment because of the methodological differences of the included studies but the high number of not applicable or irrelevant questions prompted us to evaluate the risk of bias with a tool designed for prevalence estimation. We were not able to perform subgroup analyses due to the low number of sufficiently detailed articles.

### 2.1. Data Sources and Search Strategy

We ran a systematic literature search from inception to 6 October 2019 in five electronic databases, namely, MEDLINE (via PubMed), Embase, Cochrane Register of Controlled Trials (CENTRAL), Scopus, and Web of Science. Our search query consisted of two domains describing AIDs and CF, which were connected with the ‘AND’ Boolean operator (for the full-length search key, see text, Table S2). Keywords related to AIDs were retrieved from the Connecticut-based Autoimmune Registry [23]. Since the CFTR gene and its role in the pathogenesis of CF were discovered in 1989 [24,25,26], papers published before that date were excluded; otherwise, no restrictions were imposed on the search. In addition, a manual search was performed in cited and citing papers (via Google Scholar) of the included studies and relevant reviews.

### 2.2. Selection and Eligibility

Eligible papers discussed CF patients and reported the prevalence of AIDs. Conference abstracts were included as well. Case series (>10 CF patients) and comparative studies with primary data were eligible. We excluded publications which discussed overselected CF populations (e.g., only CF patients with CF-related diabetes were included), except studies discussing specific age groups. The search yield was combined with reference management software (EndNote X9; Clarivate Analytics, Philadelphia, PA, USA). After removing duplicates, we screened the articles on the basis of title, abstract, and full text by two independent investigators (M.I. and F.D.). Any debate was resolved by third-party arbitration.

### 2.3. Data Extraction and Quality Assessment

We designed data collection sheets for each AID (a list of data extracted is presented in Table S3). Data extraction was conducted separately by two independent investigators (M.I. and F.D.). Any debate was resolved by third-party arbitration.

Two authors (M.I. and F.D.) assessed the quality of the studies by using a tool specifically designed for prevalence studies by The Joanna Briggs Institute [27]. We omitted three items from this tool because of inapplicability to our question. The results of the assessment were not incorporated into the statistical analysis but discussed in detail narratively (see Table S4, which contains our evaluation methods for the applicable domains).

### 2.4. Statistical Analysis

Meta-analytical calculations were carried out with the Comprehensive Meta-Analysis Software (CMA) version 3 (Biostat, Inc., Englewood, NJ, USA). Pooled prevalences with 95% confidence intervals (CI) were calculated. A random-effects model was used with the assumption that the prevalence of AIDs may be affected by geographical region. The CI of point estimates was calculated with the Wilson score interval model of the binomial proportions CI calculation. Statistical heterogeneity was quantified with I^2^, where 0–40% represented not important between-study heterogeneity, 30–60% was moderate, 50–90% was substantial, and 75–100% was considerable [28]; the probability was tested with chi^2^ tests (*p* = 0.1). Publication bias was estimated with funnel plots since statistical tests are not applicable to analyses with <10 studies.

The number of eligible studies allowed us to conduct a meta-analysis on the prevalence of CeD exclusively, while we summarized all the findings on other AIDs narratively.

### 2.5. Subgroups in Celiac Disease

Studies were separated in an analysis based on case, finding strategies applied to two groups as follows: studies that conducted systematic screening of the whole population prospectively (the “consecutive” group) or that which did not do so (the “non-consecutive” group). In the latter group, data were collected from medical charts retrospectively. We also investigated (1) seroprevalence for anti-tissue transglutaminase IgA antibodies (TGA-IgA), (2) seroprevalence for double positivity for TGA-IgA and anti-endomysial IgA (EMA-IgA), and (3) biopsy-verified prevalence of CeD.

## 3. Results

### 3.1. Search and Selection

**Figure 1:**
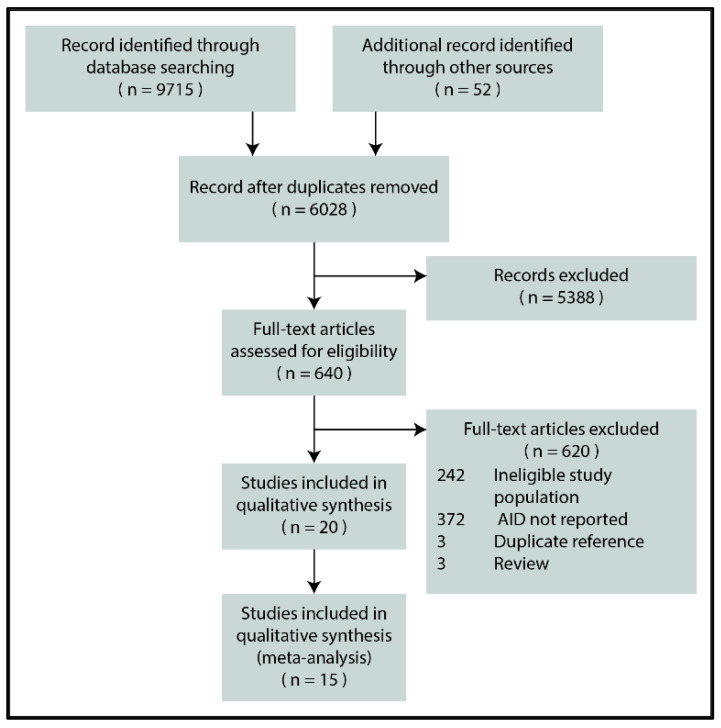
PRISMA flowchart (AID: autoimmune disease).

Figure 1 depicts the flowchart for the selection process. In the end, 20 reports were eligible for inclusion [29,30,31,32,33,34,35,36,37,38,39,40,41,42,43,44,45,46,47,48], 15 of which reporting the biopsy-verified prevalence or seroprevalence of CeD were included in the quantitative synthesis [29,30,31,32,33,34,35,36,37,38,39,40,41,42,43].

### 3.2. Celiac Disease

#### 3.2.1. Characteristics of the Studies Included

The diagnosis of CF was based on a sweat chloride test with or without genetic testing in all the studies. Six [31,32,34,36,41,43] and eight [29,30,33,37,38,39,40,42] studies were assigned to the consecutive and non-consecutive screening groups (detailed in Table 1 and Table 2), respectively. One article [35] entailed serological screening without identifying any CeD cases (zero events). Twelve studies reported data from Europe, two from Brazil [33], and one from Canada [32]. Among the consecutive studies, one reported data on adults and two on children, while all the others investigated mixed-study populations. With one exception, the non-consecutive studies included children only.

In the consecutive studies, the diagnostic workout was as follows: patients were tested with serology (detailed in Table 3), then seropositive patients underwent an upper GI endoscopy with a small bowel biopsy. Not all the non-consecutive studies mentioned the exact diagnostic workout of CeD. Prevalence of biopsy-verified CeD ranged from 0.0 to 3.5% in the consecutive screening group and 0.4 to 8.6% in the non-consecutive group.

IgA deficiency was only reported in three out of the six consecutive studies with a prevalence of 1.8% (5/282 cases) [43], 2.2% (17/790 cases) [34], and 2.6% (3/114 cases) [32].

#### 3.2.2. Results of Meta-Analysis

**Figure 2:**
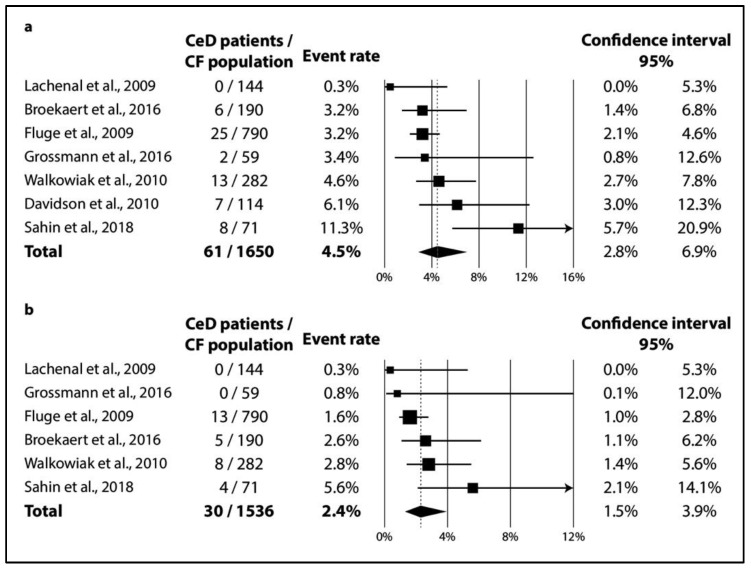
Seroprevalence of CeD: (**a**) TGA-IgA positivity; (**b**) TGA-IgA+EMA-IgA double positivity (CeD: celiac disease, CF: cystic fibrosis).

**Figure 3:**
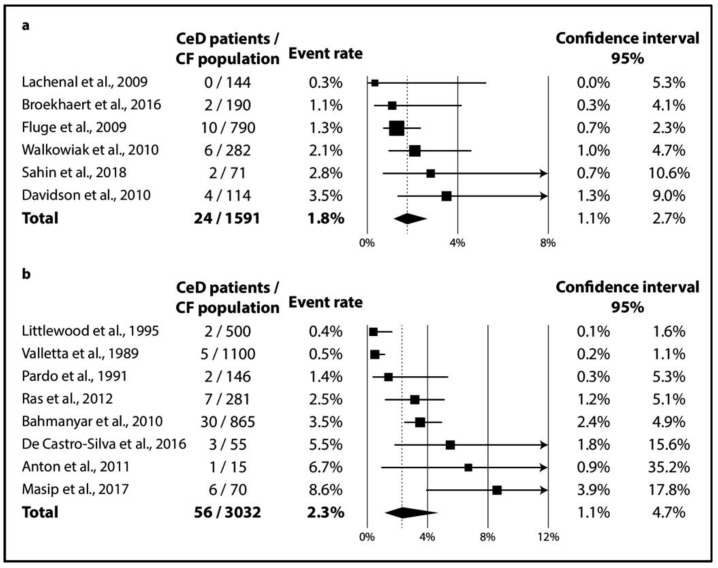
Prevalence of biopsy-verified CeD: (**a**) CeD prevalence from consecutive studies; (**b**) CeD prevalence from non-consecutive studies (CeD: celiac disease, CF: cystic fibrosis).

In CF, TGA positivity was 4.5% (CI 2.8–6.9%; I^2^ = 60% with *p* = 0.020) (Figure 2a), while TGA and EMA double positivity was 2.4% (CI 1.5–3.9%; I^2^ = 34% with *p* = 0.182) (Figure 2b). As regards biopsy-verified CeD, the six consecutive studies included a total of 1591 CF patients with a pooled prevalence of 1.8% (CI 1.1–2.7%) in a homogeneous dataset (I^2^ = 11% with *p* = 0.348) (Figure 3a). In the non-consecutive group, pooled prevalence was 2.3% (CI 1.1–4.7%; I^2^ = 81% with *p* < 0.001) on the basis of an analysis of 3032 cases (Figure 3b).

#### 3.2.3. Publication Bias

On the basis of a visual inspection of the funnel plots constructed out of a limited number of studies, we found that publication bias was unlikely to affect estimates (see Figure S1, which contains the funnel plots).

### 3.3. Systematic Review on Autoimmune Diseases Other Than Celiac Disease

Evidence from AIDs other than CeD is modest and should be treated with caution. Kesler et al. conducted a propensity-matched case–control study, including 60,055 CF patients from an ICD coding-based inpatient registry in the United States. A total of 380 suffered from Crohn’s disease, and 105 patients had ulcerative colitis [44]. Further, seven studies reported only nine cases of Crohn’s disease and seven cases of ulcerative colitis in total [30,32,36,37,45,46,47]. Lachenal et al. investigated a wide range of antibodies in the sera of 144 CF patients and found that 113 were positive for at least one antibody. ANCA, ASCA, and ANA positivity was present in >20% of the study population (none were positive for TGA), while three patients had AID (one case each for Crohn’s disease, systemic lupus erythematosus, and rheumatoid arthritis associated with autoimmune adrenal insufficiency) [36]. Strandvik et al. reported four patients (out of 102) who had undergone endoscopic retrograde cholangiopancreatography due to signs and symptoms pointing to sclerosing cholangitis, but histology did not convincingly confirm the diagnosis of primary sclerosing cholangitis [48]. Sowa et al. found no cases of inflammatory bowel disease or autoimmune hepatitis among 95 CF patients in a case–control study [46].

### 3.4. Quality Assessment

**Figure 4:**
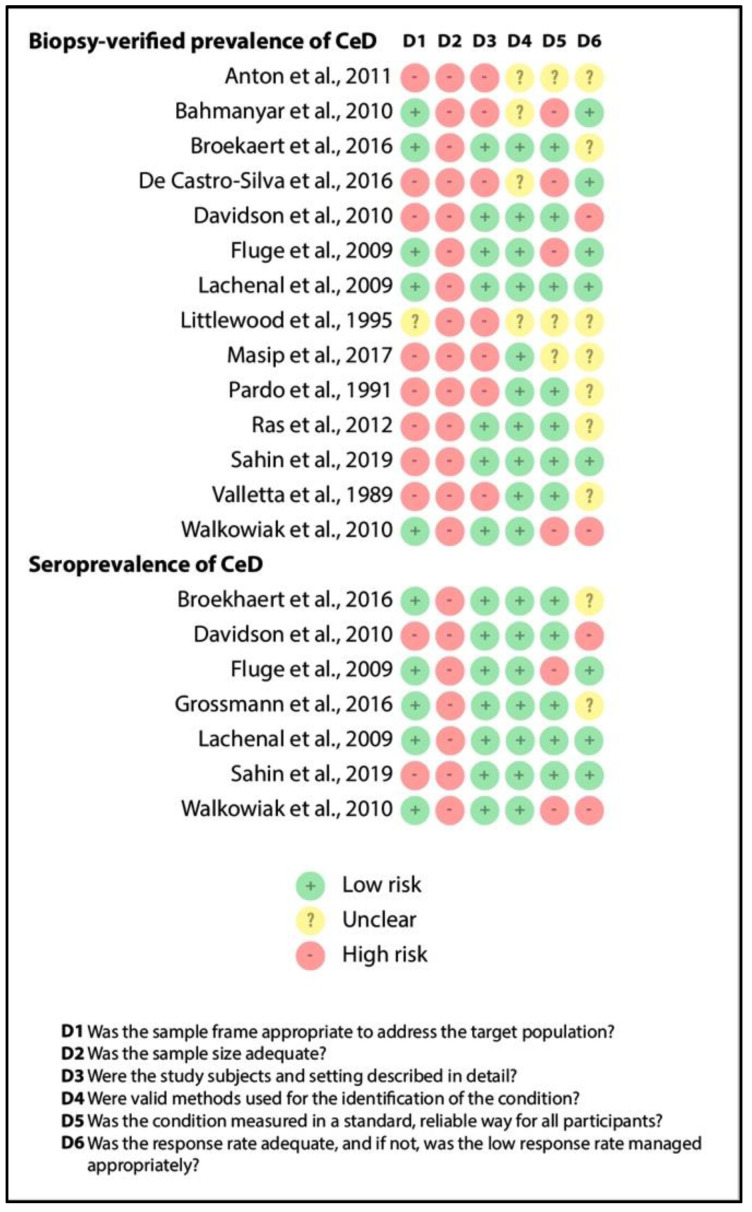
Result of the risk of bias assessment (CeD: celiac disease).

Figure 4 depicts the results of quality assessment (item by item) for biopsy-verified prevalence and seroprevalence of CeD. Although we excluded studies recruiting overselected CF populations, representativity was judged to be unsatisfactory in studies involving only pediatric or adult CF cases (see item D1). All the studies had a small sample size (imprecision, as shown by item D2). The reporting of the characteristics of the population was adequate in more than half of the studies (see item D3), as were the case definitions of CF and CeD (see items D4 and D5). Missing cases were rather problematic in the retrospective studies (see item D6). All in all, studies in the consecutive group were of better quality than that in the non-consecutive group, which was probably due to the difference in study design (retrospective vs. prospective studies).

The findings reported in the systematic review section should not be considered conclusive due to the extremely limited evidence (all items were judged as high-risk).

## 4. Discussion

### 4.1. Summary of Findings

In our meta-analysis, we planned to mathematically synthesize data on several AIDs in CF with a systematic review (as pre-specified in the protocol, see PROSPERO). Reviewing the data, we realized that only prevalence data on CeD can be aggregated with a meta-analysis. We provided evidence that 1 out of 55 CF patients had biopsy-verified CeD (Figure 3), which was more than twice as high as the global biopsy-verified CeD prevalence (1 out of 142 patients based on Singh et al. [49]). We narratively synthesized reports on other AIDs to adhere to our a priori protocol.

### 4.2. Explanation and Elaboration

Multiple mechanisms have been proposed that support immune dysregulation in CF. These include but are not limited to lipopolysaccharide hypersensitivity of alveolar macrophages [50,51]; enhanced signal transduction via NF-κB and MAPK pathways [50,52]; altered LPS-induced metabolic pathways [52]; impaired apoptosis of neutrophil granulocytes [53,54]; and an increased release of various proinflammatory cytokines [50,55], including IL-17 [56,57] and IL-6 [58,59], known as key contributors in the development of AIDs [60]. Genetic links between CF and autoimmunity should be highlighted as well [61].

The functions of the CFTR protein might be impaired in CeD, which can trigger a shift towards a pro-inflammatory state. CFTR inhibition by a gluten/gliadin-derived peptide (P31–43) activates TGA-2 while inactivating Benclin-1, thereby compromising autophagy and, through the NF-κB pathway, increasing the production of proinflammatory cytokines. The CFTR protein was thus proposed to be a “stress sensor”, activated by gliadin [62].

Coinheritance of CeD-related HLA genotypes (that is, DQ2 and DQ8) might contribute to the explanation of the pathomechanistic link between CeD and CF; however, recent evidence rather contradicts this theory. HLA-DQ2 positivity was found in 33 vs. 32% by Broekhaert et al. [31] and 25 vs. 29% [43] by Walkowiak et al. in the CF and non-CF populations, respectively. The results were consistent for HLA-DQ8 [31,43].

Clinically, most (mainly asymptomatic) CeD cases remain unrecognized even in the general population (see, the “celiac iceberg” model) [63]. The diagnostic workout might be more difficult in CF because patients tend to develop exocrine pancreatic insufficiency in up to 85% of cases [64], interfering with the “classical” symptoms of CeD. All in all, the “celiac iceberg” model seems applicable in CF as well: in the studies that performed consecutive screening, 16 out of the 24 CeD cases (67%) were newly diagnosed (see Table 1). In contrast, consecutive and non-consecutive studies did not detect a substantial difference in the prevalence of CeD (1.8% (CI 1.1–2.7%) vs. 2.3% (CI 1.1–4.7%), respectively; see Figure 3). Of note, the heterogeneity of the dataset of the previous screening strategy might not be important (I^2^ = 11% for the consecutive studies vs. I^2^ = 81% for the non-consecutive ones), revealing a potential source of heterogeneity.

The most important celiac-specific antibody is TGA-IgA, which is highly sensitive and specific to the clinical diagnosis of CeD (>95% for both) [18,20,21,65,66]. Singh et al. found the global seroprevalence of CeD (defined as TGA-IgA and/or EMA-IgA positivity) to be 1.4% (CI 1.1–1.7%). This is approximately three times lower than that estimated for TGA-IgA positivity based on consecutive screening in our meta-analysis on CF patients (4.5% (CI 2.8–6.9%)). During the diagnostic workout, total IgA should be measured because, on the basis of the results of three studies in our meta-analysis, approximately 1 out of 50 CF patients has IgA deficiency [32,34,43], which is a magnitude higher than what is expected in the general population [67,68]. In most of the studies with seroprevalence, patients with IgA deficiency were tested for positivity of one or more celiac-specific IgG antibodies. In two cases, we had no information on further investigation into IgA deficiency [35,36], and in one study [32], the diagnosis of celiac disease in these patients was ruled out by the absence of celiac-associated HLA haplotypes.

As regards the other AIDs, all are less common than CeD, while the reports identified included only a limited number of CF patients, except in one paper that reported on IBD. In this paper, out of 60,000 CF patients, IBD prevalence was 0.81% (CI 0.74–0.88%), in contrast to 0.3% prevalence in the non-CF population [69]. Crohn’s disease and ulcerative colitis had a prevalence of 0.63% (CI 0.57–0.70%) and 0.17% (CI 0.14–0.21%), respectively [44]. We must note that these data came from a multi-center survey without exact details on diagnostic strategies and therefore should be treated with caution. Due to the imbalance of the sample sizes for the studies (60,000 patients in this study vs. <5000 patients in all the other studies), we decided not to conduct a meta-analysis. The sporadic reports on autoimmune hepatitis, primary sclerosing cholangitis, and systemic lupus erythematosus warrant further investigation to assess their true prevalence.

### 4.3. Strengths and Limitations

In addition to the rigorous methodology, the main strength of this meta-analysis is its precision (i.e., statistical power). After aggregating data on more than 1500 CF patients screened through an active case-finding strategy, we found the precision of our estimates to be adequate (as reflected by the narrow confidence intervals), even if we chose to apply the random-effects model due to the expected variance in the characteristics of the study populations and disease definitions. Homogeneity in the analysis from consecutive studies strengthens the quality of evidence (Figure 3).

Unfortunately, in CeD, the number of studies from different age groups did not allow us to conduct a subgroup analysis—as shown in Table 1 and Table 2, studies reported on children, adults, or mixed populations (see item D1 in Figure 4). According to Singh et al., the prevalence of CeD is significantly higher in children than in adults [49]. This observation is consistent with the results from the consecutive studies (Table 1). Of note, aggregating data from different age groups did not increase heterogeneity considerably (I^2^ = 11%).

Data were lacking or were very limited on many AIDs. Although we aimed to include studies on type 1 diabetes mellitus and rheumatoid arthritis, we did not in the end because the papers used such fluid definitions that we were unable to separate them from CF-related diabetes and arthropathy.

After summarizing the screening results of 1591 CF patients, we identified 24 patients with biopsy-verified celiac disease. This number of patients results in a slightly imprecise estimate, as can be seen from the width of the confidence interval. A higher number of patients is needed to estimate the prevalence more accurately.

We calculated raw CeD prevalence and compared it to data already published by Singh et al. instead of calculating odds ratios or standardized incidence rates at the level of individual studies because most of the papers included lacked control groups, except for two. One study from the non-consecutive group took a sample from the Swedish Inpatient Registry: the prevalence of CeD was 3.47% (CI 2.44–4.91%) in CF vs. 0.19% (CI 0.11–0.31%) in the matched control group [30]. Another study from the consecutive group found the prevalence of CeD to be 2.82% (CI 0.78–9.70%) in CF vs. zero cases in the control group [41], although the prevalence of CeD is about 0.47% among Turkish children [70].

Concerns may also arise about the effect of regional, and age- and classification-related variance (indirectness) as potential causes of heterogeneity (see items D4 and D5 in Figure 4). As shown in Table 1 and Table 2, our conclusions are rather generalizable for Caucasians.

## 5. Conclusions

### 5.1. Implications for Practice

Our results are only conclusive for CeD and show that approximately 1 out of 22 CF patients were positive for TGA-IgA, 1 out of 40 were positive for TGA-IgA plus EMA-IgA, and 1 out of 55 had biopsy-verified CeD, numbers that are considerably higher than those measured in the general population. These results support screening for CeD in CF and raise the idea of incorporating CF into the high-risk conditions of CeD.

### 5.2. Implications for Research and Cost-Effectiveness

(1) Controlled studies should confirm our conclusions on the prevalence of CeD. (2) Screening for CeD in the general population was proven to not be cost-effective, and therefore screening among CF patients should be investigated from this perspective. (3) The clinical phenotype of CF with and without CeD should be described in detail to define those who might benefit from CeD screening and, later, from the gluten-free diet. (4) CF patient registries should place more emphasis on recording AIDs and, conversely, AID registries should focus on CF.

## Figures and Tables

**Table 1:** CeD prevalence from consecutive studies (SD: standard deviation, CF: cystic fibrosis, CeD: celiac disease).

		Lachenal et al., 2009 [36]	Broekaert et al., 2016 [31]	Fluge et al., 2009 [34]	Walkowiak et al., 2010 [43]	Sahin et al., 2019 [41]	Davidson et al., 2009 [32]
Setting							
	Country (centers)	France (Lyon)	Germany (Cologne)	Denmark, Norway, and Sweden	Poland	Turkey (Istanbul)	Canada (Vancouver)
	Recruitment period	Jan 2000–Jan 2007	N/A	2004–2005	2006–2009	Oct 2015–Mar 2017	N/A
Prospective		yes	yes	yes	yes	yes	yes
Age group		adults	adults and children	adults and children	adults and children	children	children
Age (mean (SD)		25 (5.9)	25.5 (19.6)	N/A	17.3 (11.3)	9.9 (5.5)	N/A
Total number of patients with CF		144	190	790	282	71	114
Number of patients with known CeD		0 (0.0%)	0 (0.0%)	6 (0.8%)	2 (0.7%)	0 (0.0%)	0 (0.0%)
Number of patients with de novo diagnosed CeD		0 (0.0%)	2 (1.1%)	4 (0.5%)	4 (1.4%)	2 (2.8%)	4 (3.5%)
Total prevalence of CeD		0/144 (0.0%)	2/190 (1.1%)	10/790 (1.3%)	6/282 (2.1%)	2/71 (2.8%)	4/114 (3.5%)
Diagnostic criteria of CF		sweat test + genetics	sweat test + genetics	N/A	sweat test + genetics	sweat test + genetics	N/A
Diagnostic criteria of CeD		serology	histology	histology	histology	histology	histology

**Table 2:** CeD prevalence from non-consecutive studies (SD: standard deviation, CF: cystic fibrosis, CeD: celiac disease).

		Littlewood et al., 1995 [37]	Valletta et al., 1989 [42]	Pardo et al., 1991 [39]	Ras et al., 2012 [40]	Bahmanyar et al., 2010 [30]	De Castro et al., 2016 [33]	Anton et al., 2011 [29]	Masip et al., 2017 [38]
Setting									
	Country (centers)	UK (Leeds)	Italy (Verona)	Italy (Palermo)	the Netherlands	Sweden	Brazil (Fortaleza)	Romania (Iasi)	Spain (Valencia)
	Recruitment period	N/A	N/A	N/A	N/A	1968–2003	N/A	N/A	2005–2015
Prospective		no	yes	no	yes	no	no	no	no
Age group		children	children	children	children	adults and children	children	children	children
Age (mean (SD)		N/A	N/A	N/A	10 (5.2)	N/A	4.7 (4.3)	N/A	N/A
Total number of patients with CF		500	1100	146	281	865	55	15	70
Total prevalence of CeD		2/500 (0.4%)	5/1100 (0.5%)	2/146 (1.4%)	7/281 (2.5%)	30/865 (3.5%)	3/55 (5.5%)	1/15 (6.7%)	6/70 (8.6%)
Diagnostic criteria of CF		N/A	sweat test	sweat test	sweat test + genetics	sweat test + genetics	N/A	N/A	sweat test + genetics
Diagnostic criteria of CeD		N/A	histology	histology	ESPGHAN	N/A	N/A	N/A	ESPGHAN

**Table 3:** CeD seroprevalence (SD: standard deviation, CF: cystic fibrosis, IIF: indirect immunofluorescence).

		Lachenal et al., 2009 [36]	Grossmann et al., 2016 [35]	Broekaert et al., 2016 [31]	Fluge et al., 2009 [34]	Walkowiak et al., 2010 [43]	Sahin et al., 2019 [41]	Davidson et al., 2009 [32]
Setting								
	Country (centers)	France (Lyon)	Germany (Dresden)	Germany (Cologne)	Denmark, Norway, and Sweden	Poland	Turkey (Istanbul)	Canada (Vancouver)
	Recruitment period	Jan 2000– Jan 2007	N/A	N/A	2004–2005	2006–2009	Oct 2015– Mar 2017	N/A
Prospective		yes	yes	yes	yes	yes	yes	yes
Age (mean (SD))		25 (5.9)	12 (17)	25.5 (19.6)	N/A	17.3 (11.3)	9.9 (5.5)	N/A
Total number of patients with CF		144	59	190	790	282	71	114
TGA-IgA positivity								
	%	0.0% (0)	3.4% (2)	3.2% (6)	N/A	3.9% (11)	11.3% (8)	6.1% (2)
	Method	ELISA	ELISA	ELISA	ELISA	nephelometry	ELISA	N/A
	Cut-off	N/A	3.91 U/ml	20 U/ml	50 U/ml	N/A	N/A	N/A
EMA-IgA positivity								
	%	0% (0)	0% (0)	3.2% (6)	0.9% (7)	N/A	N/A	N/A
	Method	IIF (monkey esophagus)	IIF (monkey esophagus)	IIF (monkey liver)	IIF (monkey esophagus)	N/A	N/A	N/A
	Cut-off	N/A	1:10	1:10	1:5	N/A	N/A	N/A
TGA-IgA and EMA-IgA positivity		0% (0)	0% (0)	2.6% (5)	0.9% (7)	2.1% (6)	5.6% (4)	N/A
IgA deficiency		N/A	N/A	N/A	2.2% (17)	1.8% (5)	N/A	2.6% (3)

## Data Availability

Data are contained within the article or supplementary material.

## Supplementary Materials

The following are available online at https://www.mdpi.com/article/10.3390/jpm11090859/s1, Figure S1: Funnel plots ((a) celiac disease prevalence from consecutive studies; (b) celiac disease prevalence from non-consecutive studies; (c) TGA-IgA positivity; (d) TGA-IgA+EMA-IgA double-positivity). Table S1: PRISMA 2009 Checklist. Table S2: Search query. Table S3: Headers of the data collection sheet.
