# Supplementary material for: Increased Prevalence of Celiac Disease in Patients with Cystic Fibrosis: A Systematic Review and Meta-Analysis
Source: J Pers Med. 2021 Aug 28;11(9):859. doi: 10.3390/jpm11090859 (PMC8470465; doi:10.3390/jpm11090859)

Table S1: PRISMA 2009 Checklist

Section/topic	#	Checklist item	Reported on page #
**TITLE**			
Title	1	Identify the report as a systematic review, meta-analysis, or both.	1
**ABSTRACT**			
Structured summary	2	Provide a structured summary including, as applicable: background; objectives; data sources; study eligibility criteria, participants, and interventions; study appraisal and synthesis methods; results; limitations; conclusions and implications of key findings; systematic review registration number.	1
**INTRODUCTION**			
Rationale	3	Describe the rationale for the review in the context of what is already known.	2
Objectives	4	Provide an explicit statement of questions being addressed with reference to participants, interventions, comparisons, outcomes, and study design (PICOS).	2
**METHODS**			
Protocol and registration	5	Indicate if a review protocol exists, if and where it can be accessed (e.g., Web address), and, if available, provide registration information including registration number.	2
Eligibility criteria	6	Specify study characteristics (e.g., PICOS, length of follow-up) and report characteristics (e.g., years considered, language, publication status) used as criteria for eligibility, giving rationale.	3
Information sources	7	Describe all information sources (e.g., databases with dates of coverage, contact with study authors to identify additional studies) in the search and date last searched.	3
Search	8	Present full electronic search strategy for at least one database, including any limits used, such that it could be repeated.	3
Study selection	9	State the process for selecting studies (i.e., screening, eligibility, included in systematic review, and, if applicable, included in the meta-analysis).	3
Data collection process	10	Describe method of data extraction from reports (e.g., piloted forms, independently, in duplicate) and any processes for obtaining and confirming data from investigators.	3
Data items	11	List and define all variables for which data were sought (e.g., PICOS, funding sources) and any assumptions and simplifications made.	3
Risk of bias in individual studies	12	Describe methods used for assessing risk of bias of individual studies (including specification of whether this was done at the study or outcome level), and how this information is to be used in any data synthesis.	3
Summary measures	13	State the principal summary measures (e.g., risk ratio, difference in means).	3
Synthesis of results	14	Describe the methods of handling data and combining results of studies, if done, including measures of consistency (e.g., I^2) for each meta-analysis.	3

Section/topic	#	Checklist item	Reported on page #
Risk of bias across studies	15	Specify any assessment of risk of bias that may affect the cumulative evidence (e.g., publication bias, selective reporting within studies).	3
Additional analyses	16	Describe methods of additional analyses (e.g., sensitivity or subgroup analyses, meta-regression), if done, indicating which were pre-specified.	-
**RESULTS**			
Study selection	17	Give numbers of studies screened, assessed for eligibility, and included in the review, with reasons for exclusions at each stage, ideally with a flow diagram.	4
Study characteristics	18	For each study, present characteristics for which data were extracted (e.g., study size, PICOS, follow-up period) and provide the citations.	4
Risk of bias within studies	19	Present data on risk of bias of each study and, if available, any outcome level assessment (see item 12).	7
Results of individual studies	20	For all outcomes considered (benefits or harms), present, for each study: (a) simple summary data for each intervention group (b) effect estimates and confidence intervals, ideally with a forest plot.	5
Synthesis of results	21	Present results of each meta-analysis done, including confidence intervals and measures of consistency.	5
Risk of bias across studies	22	Present results of any assessment of risk of bias across studies (see Item 15).	6
Additional analysis	23	Give results of additional analyses, if done (e.g., sensitivity or subgroup analyses, meta-regression [see Item 16]).	-
**DISCUSSION**			
Summary of evidence	24	Summarize the main findings including the strength of evidence for each main outcome; consider their relevance to key groups (e.g., healthcare providers, users, and policy makers).	8
Limitations	25	Discuss limitations at study and outcome level (e.g., risk of bias), and at review-level (e.g., incomplete retrieval of identified research, reporting bias).	9
Conclusions	26	Provide a general interpretation of the results in the context of other evidence, and implications for future research.	10
**FUNDING**			
Funding	27	Describe sources of funding for the systematic review and other support (e.g., supply of data); role of funders for the systematic review.	10

From: Moher D, Liberati A, Tetzlaff J, Altman DG, The PRISMA Group (2009). Preferred Reporting Items for Systematic Reviews and Meta-Analyses: The PRISMA Statement. PLoS Med 6(7): e1000097. doi:10.1371/journal.pmed1000097

For more information, visit: **www.prisma-statement.org**.

Table S2: Search query

**Embase (filter: ":ab,ti"), Scopus (filter: "TITLE-ABS-KEY"), Central (filter: none), Web of Science (filter: none)**	("cystic fibrosis" or "CFTR")
	AND
	(("ibd" or "inflammatory bowel disease" or "crohn" or "crohn's" or "crohns" or "ulcerative colitis") or ("celiac" or "coeliac" or "gluten" or "gliadin") or ("Rheumatoid arthritis") or ("diabetes" and ("type 1" or "autoimmune")) or ("Primary sclerosing cholangitis" or "psc") or ("multiple sclerosis") or ("Systemic lupus erythematosus" or "sle") or ("hemophilia A" or "haemophilia A") or ("pbc" or "Primary biliary cirrhosis" or "Primary biliary cholangitis") or ("autoimmune pancreatitis") or ("autoimmune hepatitis") or ("graves'" or "graves disease" or "basedow" or "basedow's" or "basedows") or ("scleroderma") or ("morvan" or "fibrillary chorea") or ("kawasaki" or "Mucocutaneous Lymph Node Syndrome") or ("sjogren's" or "sjorgens" or "sjogren" or "sicca syndrome") or ("Churg-Strauss") or ("Wegener granulomatosis") or ("Myasthenia gravis") or ("Hashimoto" or "hashimoto's" or "hashimotos") or ("polymyositis" or "dermatomyositis") or ("addison's" or "addisons" or "addison" or "hypoadrenocorticism") or ("Behcets" or "Behcet's" or "Behcet") or ("Guillain-Barre syndrome") or ("atrophic gastritis" or "pernicious anemia" or "atropic gastritis" or "pernicious anemia") or ("Autoimmune hemolytic anemia") or ("rheumatic fever") or (("hashimoto" or "hashimoto's" or "hashimotos") and ("encephalopathy" or "encephalitis")) or ("Polyarteritis Nodosa") or ("Goodpasture" or "goodpasture's" or "goodpastures") or ("ipex") or ("Antiphospholipid syndrome") or ("Mixed connective tissue disease") or ("Microscopic polyangiitis") or ("cogan") or ("polychondritis") or ("Isaacs'" or "isaac's" or "neuromyotonia") or ("vitiligo") or ("batten disease" or "Neuronal Ceroid Lipofuscinoses") or ("Pemphigus") or ("still's disease" or "stills disease" or "still disease") or ("autoimmune polyglandular syndrome" or "apeced") or ("takayasu" or "takayasu's" or "takayasus" or "aortic arch syndrome") or ("bullous pemphigoid") or ("Autoimmune disseminated encephalomyelitis" or "Autoimmune encephalomyelitis" or "disseminated encephalomyelitis") or ("Autoimmune Lymphoproliferative Syndrome") or ("poems") or ("Alopecia areata") or ("pandas") or ("Rasmussen's" or "Rasmussens") or ("stiff-person" or "stiff-man") or ("CREST syndrome") or ("hla-b27" and "uveitis") or ("Felty's syndrome" or "Feltys syndrome" or "Felty Syndrome") or ("Autoimmune neutropenia") or ("Epidermolysis Bullosa Acquisita") or ("evans syndrome") or ("Immune thrombocytopenic purpura") or ("Essential mixed cryoglobulinemia") or ("Erythema elevatum diutinum") or ("Sympathetic ophthalmia") or ("Discoid Lupus Erthematosus") or ("linear morphea") or ("Cicatricial pemphigoid") or ("Autoimmune hypoparathyroidism") or ("Autoimmune hypophysitis ") or ("Autoimmune oophoritis") or ("Autoimmune orchitis") or ("Encephalitis lethargica") or ("miller fisher") or ("Vogt-Koyanagi-Harada" or

	"uveomeningitis" or "uveomeningoencephalitic") or ("autoimmunity" or "autoimmune" or "immune mediated"))
**MEDLINE (filter: none)**	("cystic fibrosis" or "CFTR" or "Cystic Fibrosis"[Mesh])
	AND
	(("Inflammatory Bowel Diseases"[Mesh] or "ibd" or "inflammatory bowel disease" or "crohn" or "crohn's" or "crohns" or "ulcerative colitis") or ("celiac" or "coeliac" or "gluten" or "gliadin" or "Celiac Disease"[Mesh] or "Glutens"[Mesh] or "Gliadin"[Mesh]) or ("Rheumatoid arthritis" or "Arthritis, Rheumatoid"[Mesh]) or ("diabetes" and ("type 1" or "autoimmune") or "Diabetes Mellitus, Type 1"[Mesh]) or ("Primary sclerosing cholangitis" or "psc" or "Cholangitis, Sclerosing"[Mesh]) or ("multiple sclerosis" or "Multiple Sclerosis"[Mesh]) or ("Systemic lupus erythematosus" or "sle" or "Lupus Erythematosus, Systemic"[Mesh]) or ("hemophilia A" or "haemophilia A" or "Hemophilia A"[Mesh]) or ("pbc" or "Primary biliary cirrhosis" or "Primary biliary cholangitis") or ("autoimmune pancreatitis") or ("autoimmune hepatitis" or "Hepatitis, Autoimmune"[Mesh]) or ("graves'" or "graves disease" or "basedow" or "basedow's" or "basedows" or "Graves Disease"[Mesh]) or ("scleroderma" or "Scleroderma, Systemic"[Mesh] OR "Scleroderma, Localized"[Mesh]) or ("morvan" or "fibrillary chorea") or ("kawasaki" or "Mucocutaneous Lymph Node Syndrome" or "Mucocutaneous Lymph Node Syndrome"[Mesh]) or ("sjogren's" or "sjorgens" or "sjogren" or "sicca syndrome" or "Sjogren's Syndrome"[Mesh]) or ("Churg-Strauss" or "Churg-Strauss Syndrome"[Mesh]) or ("Wegener granulomatosis" or "Granulomatosis with Polyangiitis"[Mesh]) or ("Myasthenia gravis" or "Myasthenia Gravis"[Mesh]) or ("Hashimoto" or "hashimoto's" or "hashimotos" or "Hashimoto Disease"[Mesh]) or ("polymyositis" or "dermatomyositis" or "Polymyositis"[Mesh] or "Dermatomyositis"[Mesh]) or ("addison's" or "addisons" or "addison" or "hypoadrenocorticism" or "Addison Disease"[Mesh]) or ("Behcets" or "Behcet's" or "Behcet" or "Behcet Syndrome"[Mesh]) or ("Guillain-Barre syndrome" or "Guillain-Barre Syndrome"[Mesh]) or ("atrophic gastritis" or "pernicious anemia" or "Anemia, Pernicious"[Mesh] or "atropic gastritis" or "pernicious anaemia") or ("Autoimmune hemolytic anemia" or "Anemia, Hemolytic, Autoimmune"[Mesh]) or ("rheumatic fever" or "Rheumatic Fever"[Mesh]) or (("hashimoto" or "hashimoto's" or "hashimotos") and ("encephalopathy" or "encephalitis")) or ("Polyarteritis Nodosa" or "Polyarteritis Nodosa"[Mesh]) or ("Goodpasture" or "goodpasture's" or "goodpastures") or ("ipex") or ("Antiphospholipid syndrome" or "Antiphospholipid Syndrome"[Mesh]) or ("Mixed connective tissue disease" or "Mixed Connective Tissue Disease"[Mesh]) or ("Microscopic polyangiitis" or "Microscopic Polyangiitis"[Mesh]) or ("cogan" or "Cogan Syndrome"[Mesh]) or ("polychondritis" or "Polychondritis, Relapsing"[Mesh]) or ("Isaacs'" or "isaac's" or

	"neuromyotonia" or "Isaacs Syndrome"[Mesh]) or ("vitiligo" or "Vitiligo"[Mesh]) or ("batten disease" or "Neuronal Ceroid Lipofuscinoses" or "Neuronal Ceroid-Lipofuscinoses"[Mesh]) or ("Pemphigus" or "Pemphigus"[Mesh]) or ("still's disease" or "stills disease" or "still disease" or "Still's Disease, Adult-Onset"[Mesh]) or ("autoimmune polyglandular syndrome" or "apeced") or ("takayasu" or "takayasu's" or "takayasus" or "aortic arch syndrome" or "Takayasu Arteritis"[Mesh]) or ("bullous pemphigoid" or "Pemphigoid, Bullous"[Mesh]) or ("Autoimmune disseminated encephalomyelitis" or "Autoimmune encephalomyelitis" or "disseminated encephalomyelitis" or "Encephalomyelitis, Autoimmune, Experimental"[Mesh]) or ("Autoimmune Lymphoproliferative Syndrome" or "Autoimmune Lymphoproliferative Syndrome"[Mesh]) or ("poems" or "POEMS Syndrome"[Mesh]) or ("Alopecia areata" or "Alopecia Areata"[Mesh]) or ("pandas") or ("Rasmussen's" or "Rasmussens") or ("stiff-person" or "stiff-man" or "Stiff-Person Syndrome"[Mesh]) or ("CREST syndrome" or "CREST Syndrome"[Mesh]) or ("hla-b27" and "uveitis") or ("Felty's syndrome" or "Feltys syndrome" or "Felty Syndrome" or "Felty Syndrome"[Mesh]) or ("Autoimmune neutropenia") or ("Epidermolysis Bullosa Acquisita" or "Epidermolysis Bullosa Acquisita"[Mesh]) or ("evans syndrome") or ("Immune thrombocytopenic purpura" or "Purpura, Thrombocytopenic, Idiopathic"[Mesh]) or ("Essential mixed cryoglobulinemia" or "Cryoglobulinemia, Familial Mixed" [Supplementary Concept]) or ("Erythema elevatum diutinum") or ("Sympathetic ophthalmia" or "Ophthalmia, Sympathetic"[Mesh]) or ("Discoid Lupus Erthematosus") or ("linear morphea") or ("Cicatricial pemphigoid" or "Pemphigoid, Benign Mucous Membrane"[Mesh]) or ("Autoimmune hypoparathyroidism") or ("Autoimmune hypophysitis " or "Autoimmune Hypophysitis"[Mesh]) or ("Autoimmune oophoritis") or ("Autoimmune orchitis") or ("Encephalitis lethargica" or "Parkinson Disease, Postencephalitic"[Mesh]) or ("miller fisher" or "Miller Fisher Syndrome"[Mesh]) or ("Vogt-Koyanagi-Harada" or "uveomeningitis" or "uveomeningoencephalitis" or "Uveomeningoencephalitic Syndrome"[Mesh]) or ("autoimmunity" or "autoimmune" or "immune mediated" or "autoimmune diseases"[Mesh]))

Table S3: Headers of the data collection sheet

Publication year		
Title		
Retrospective data collection (y/n)		
Case finding strategy (active/passive)		
Study CF population	Age-group	Mean
		Median
		Standard deviation

		Interquartile range
		Range
	Center(s)	
	Recruitment period	
	Gender ratio (f/m)	
	Inclusion criteria	
	Exclusion criteria	
	Dropout (patient number)	
CF diagnosis	Description	
	Sweat test (number)	
	Sweat test threshold (mmol/l)	
	Genetics (y/n)	
Diagnostic definition of AIDs included in the search strategy		
CF population (patient number)		
AID population (patient number)		

Table S4: Evaluation strategy for risk of bias assessment domains

**1. Was the sample frame appropriate to address the target population?**	We estimated every study included patents from a defined age group high risk.
**2. Were study participants recruited in an appropriate way?**	Not applicable. Random selection from the CF population is not feasible in these clinical research settings.
**3. Was the sample size adequate?**	According to the attached formula (calculated with the prevalence of CeD from the study by Singh et al.), the adequate sample size for evaluate the prevalence of biopsy-verified CeD is 2180 and for evaluate the prevalence of seroprevalence is 1082. Studies with the population less than this were judged high risk studies.
**4. Were the study subjects and setting described in detail?**	Minimal requirement for this domain is the description of the age ((median or modus) and (standard deviation or interquartile range or range)) and the gender ratio of the study population.
**5. Was data analysis conducted with sufficient coverage of the identified sample?**	Not applicable. There was not any information about this in any studies.
**6. Were valid methods used for the identification of the condition?**	We exclude every study with obviously invalid identification of the condition, but there are retrospective studies where only the medical history can be available, and the exact diagnostic procedure is unclear.

**7. Was the condition measured in a standard, reliable way for all participants?**	We judged consecutive studies with patients diagnosed with CeD before the study and every retrospective study high risk.
**8. Was there appropriate statistical analysis?**	Not applicable. We used only raw data.
**9. Was the response rate adequate, and if not, was the low response rate managed appropriately?**	Based on the Cochrane Handbook for Systematic Reviews of Interventions, we consider studies with more than 80% response rate low risk of bias.

Figure S1: Funnel plots (a. Celiac disease prevalence from consecutive studies; b. Celiac disease prevalence from non-consecutive studies; c. TGA-IgA positivity; d. TGA-IgA+EMA-IgA double-positivity)

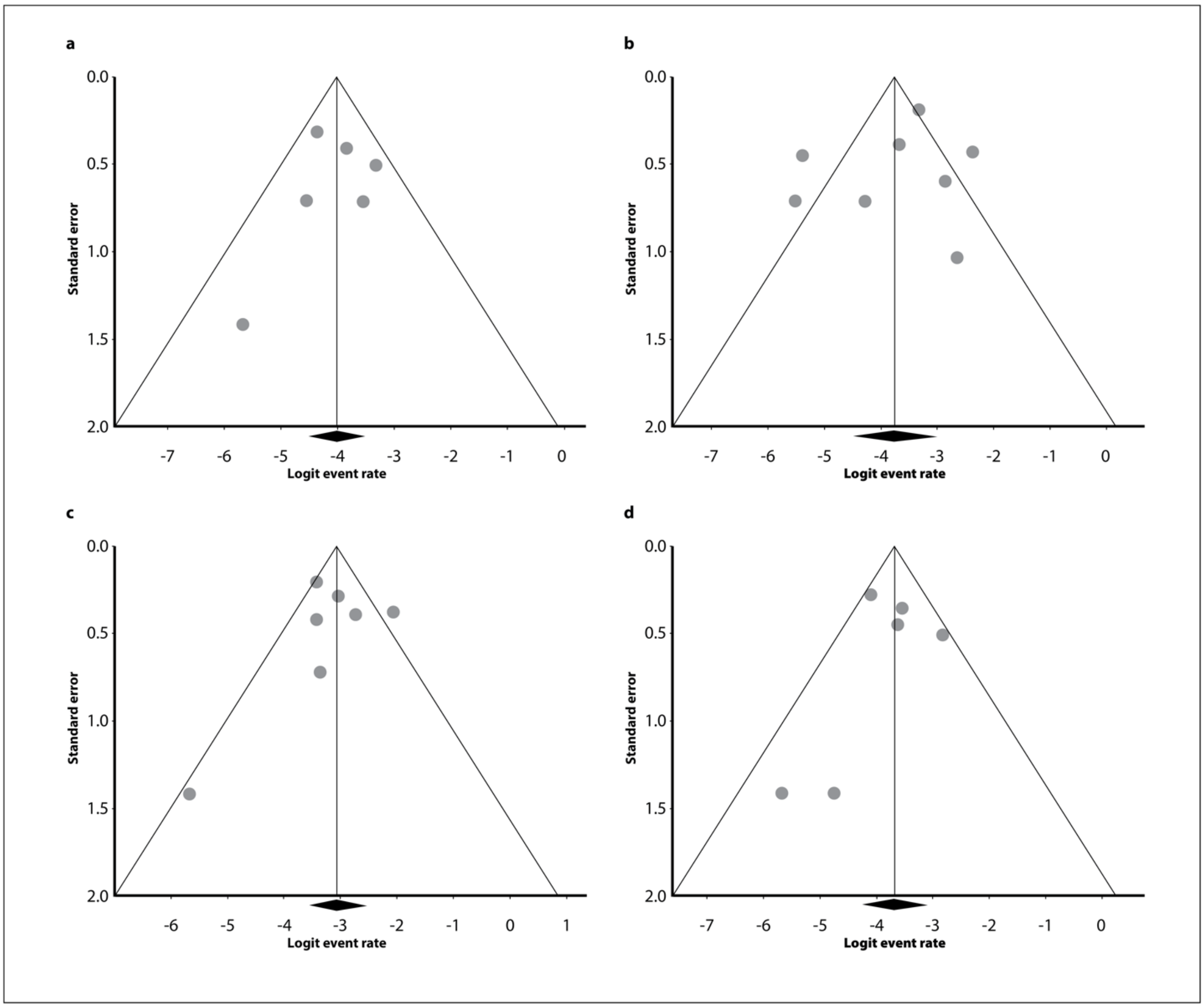

Supplement: Supplementary file 1 [file jpm-11-00859-s001.zip › jpm-1327978-supplementary.pdf]
